# Pregnancy follow-ups in Family Medicine: A retrospective study

**DOI:** 10.12669/pjms.41.7.8099

**Published:** 2025-07

**Authors:** Olgun Goktas

**Affiliations:** 1 Dr. Olgun Goktas Associate Professor, Uludag University Family Health Center, Nilufer, Bursa, Turkey

**Keywords:** Family medicine, Follow-ups, Pregnancy, Prevention, Primary health care

## Abstract

**Objective:**

To evaluate the effects and results of modern improved pregnancy follow-ups.

**Methods:**

This retrospective study was conducted with a total of 248 pregnant women between 20 to 43 years of age. In the study, all data belonging to the follow-up of pregnant women who were registered to the Family Health Center between April 1^st^, 2022, and March 31^st^, 2023, were retrieved from the database retrospectively. Sociodemographic characteristics, habits, pregnancy information and counseling process, pregnancy physical examination and laboratory findings, risk factors, drugs used, vaccinations, danger signs developing during pregnancy, and complications were determined and recorded. The data of all follow-ups were analyzed in terms of risk, disease, complications, and outcomes.

**Results:**

The age of the pregnant women was found to be 28.72±3.97 years. While the mean number of general follow-ups was 3.56±1.04 in pregnant women, the mean number of psychosocial follow-ups was 1.11±0.48. The mean weeks of gestation were found to be 36.59±7.65. It was seen that 59.5% of the pregnant women had a normal and live birth, 29.6% had a cesarean and live birth, 4.5% had an abortion, 6.1% were still pregnant and 0.4% had other result pregnancies. The number of general and psychosocial follow-ups of the pregnant women with miscarriage, abortion, and other complications during pregnancy was lower than in the other groups (p=0.01).

**Conclusion:**

Our study showed that miscarriage, abortion, and other complications are very rare with the increase in the number of general and especially psychosocial follow-ups before and during pregnancy. Pregnancy follow-ups carried out by family physicians with developed guidelines are important for the health of the mother and baby.

## INTRODUCTION

Antenatal care is important for the pregnancy period and the quality and results of the practices in different countries of the world are different. However, women value the service they will receive. The quality of health care received during pregnancy increases the level and efficiency of pregnant and infant health. A review study dealing with cultural and sociodemographic characteristics in different countries revealed that women want and need a positive pregnancy period, and they want to be followed by experienced physicians with good clinical equipment in order to avoid the risks of illness and death during pregnancy.^1^

Although progress has been made in reducing pregnancy-related diseases and mortality rates, these problems continue to increase. Countries should expand and continue these developments in line with their own health policies in this area. Before pregnancy, during pregnancy, and at the end of pregnancy, the mother and baby have the right to receive the highest quality service. The World Health Organization (WHO) recommends incentive programs to prevent pregnancy-related diseases and reduce mortality. For this purpose and in line with the Sustainable Development Goals (SDGs), it prepares up-to-date guidelines on maternal and infant health during pregnancy and recommends them to countries.^2^ For a healthy pregnancy and birth, it is important for the health of both the mother and the baby that the expectant mother goes to the doctor regularly. In a study, it was concluded that pregnant women who were under regular physician follow-up reached a higher level of knowledge and this did not change according to demographic characteristics.^3^

Along with technological developments in the world, developments in modern pregnancy follow-ups in family medicine are important. In family medicine, continuous health care with a holistic and comprehensive approach that fully evaluates the individual and the family is even more important in a sensitive period such as pregnancy. Although there is information on pregnancy follow-ups in the literature, the information on pregnancy outcomes of large, long-term, and comprehensive care services is insufficient. There is a need for region-based population data blending experiences and large-sample information. Evaluation of registered data in family medicine is important in this respect. In this study, we aimed to evaluate the effects and results of modern improved pregnancy follow-ups.

## METHODS

A total of 248 pregnant women between 20 to 43 years of age who applied to Bursa Uludağ University Family Health Center between April 1st, 2022, and March 31st, 2023 were included in this retrospective study. A simple random sampling method was used in the study. In this context, it has been observed that pregnant women who come to the Family Health Center and are under regular follow-up constitute the population and this number is N=700. In this context, it was seen that 245 pregnant women could represent the universe at a 5% error level and 95% confidence level. The study was conducted on 248 pregnant women with valid data. Pregnant women who were not registered with the family health center and left the region for any reason were not included in the study.

All information in the study was obtained from the family medicine information system. Sociodemographic characteristics, habits, pregnancy information and counseling process, pregnancy physical examination and laboratory findings, risk factors, drugs used, vaccinations, danger signs developing during pregnancy, complications, and postnatal care information of 248 registered pregnant women were determined and recorded. In addition, since improved pregnancy outcomes may result from factors such as the mother’s education level, high socioeconomic level, number of children born, the mother’s age and prior knowledge about pregnancy, information in this regard was also evaluated. The data of all follow-ups made within the framework of holistic, inclusive, and continuous care, accompanied by updated modern guidelines by the Family Health Center team, were analyzed in terms of risk, disease, complications, and outcomes. The data were obtained from the program in the Family Medicine Information Registration System, where information in standard forms used in pregnancy follow-up is stored on the computer. The data were obtained from the information in the “Pregnant Baby Child Psychosocial Monitoring Form”^4^ used in pregnancy monitoring.

Descriptive statistics were presented as mean ± standard deviation values for continuous variables, and frequency and percentage for proportional variables. The conformity of the data to the normal distribution was evaluated with the Kolmogorov-Smirnov test. Although the measurements were in accordance with the normal distribution of the data (p>0.05), non-parametric methods were used in the comparisons between the groups because the amount of data within the group was below the n=30 level. For comparisons between groups, the Mann-Whitney U test was used in two-stage groups and the Kruskall-Wallis test was used in groups of three or more. In the groups that were determined as significant, the group that made the difference was determined by the Mann-Whitney U test. The results were evaluated with the Pearson correlation test due to the normality and sufficient amount of data in the examination of the relationship between the number of follow-ups and the age, height, weight, BMI (Body Mass Index), and gestational week of the pregnants. In the study, p<0.05 was accepted as the critical decision value. The data were analyzed using the SPSS 25.0 (Statistical Packages of Social Sciences) program on the computer.

### Ethical Approval:

This retrospective study was performed after receiving the approval of the Clinical Research Ethics Committee of Bursa Uludag University (Reference no: 2023-7/17, dated: 11.04.2023) and in accordance with the Declaration of Helsinki.

## RESULTS

The age of the pregnants was found to be 28.72±3.97 years. It was determined that the height measurements were 164.61±8.99 cm and the weights were 69.02±12.00 kg. BMI levels were determined to be 26.63±21.41 levels. While the mean number of general follow-ups was 3.56±1.04 in pregnant women, the mean number of psychosocial follow-ups was 1.11±0.48. The mean weeks of gestation were found to be 36.59±7.65. (Table-III).

**Table-I:** Sociodemographic and general characteristics of pregnant women.

		n	%
Occupation	Unemployed	96	38.7%
	Working	152	61.3%
Education	High school and below	34	13.7%
	University	214	86.3%
Smoking	Never smoked	224	90.3%
	Quit during pregnancy	21	8.5%
	Current smokers	3	1.2%
Alcohol	Never used	247	9.6%
	Quit during pregnancy	1	0.4%
Abnormal vital signs	No	248	100.0%
	Yes	0	0.0%
Laboratories and tests	No	248	100.0%
	Yes	0	0.0%
Rh incompatibility	No	220	88.7%
	Yes	28	11.3%
Disease	N	174	70.2%
	1	38	15.3%
	2 and more	36	14.5%

**Table-II:** Pregnancy-related characteristics of pregnant women.

		n	%
Medication	No	177	71.4%
	Yes	71	28.6%
Other risk factors	No	184	74.2%
	1	41	16.5%
	2 and more	23	9.3%
Vitamin and/or iron supplementation	No	12	0.8%
	Yes	236	95.2%
Vaccination during pregnancy	No	70	28.2%
	Tetanus only	178	71.8%
Signs of danger during pregnancy	No	218	87.9%
	Yes	30	12.1%
Complication in pregnancy	No	239	96.4%
	Yes	9	3.6%
Mode of delivery and outcome	Normal-live birth	147	59.5%
	Live birth by cesarean	73	29.6%
	Miscarriage and abortion	11	4.5%
	Other	1	0.4%
	Pregnancy continued	15	6.1%

**Table-III:** Examination of the measurements in pregnant women.

	X±s.d
Age (Years)	28.72±3.97
Height (Centimeter-cm)	164.1±8.99
Weight (Kilogram-kg)	69.02±12
Body mass index (kg/m²)	26.63±21.41
Number of general follow-ups during pregnancy	3.56±1.04
Number of psychosocial follow-ups during pregnancy	1.11±0.48
Total week of gestation	36.59±7.65

It was also observed that 61.3% of the pregnant women were working in an income-generating job. It was observed that the education levels were high school and below with 13.7%, and undergraduate level with 86.3%. We also noted that 90.3% of the pregnant women never smoked, 8.5% quit soking during pregnancy while 1.2% were still smoking. The study showed that 99.6% of the pregnant women did not use alcohol. It was observed that there was no problem with vital signs and laboratory results in all pregnants, 11.3% had Rh incompatibility. About 70.2% of them were free from any disease while 15.3% had one disease and 14.5% had two or more diseases. Table-I Vitamin and/or iron supplementation were used by 95.2% and 28.2% were not vaccinated during pregnancy. About 12.1% of pregnant women were at risk during pregnancy while pregnancy-related complications were seen in 3.6%.

This study also showed that 59.5% of the pregnant women had a normal and live birth, 29.6% had a cesarean and live birth, 4.5% had an abortion, 6.1% were still pregnant.Table-II It was seen that the general follow-up numbers according to the professions were significantly different, The group of working women in an income-generating job had a higher number of follow-ups (p=0.03). We also noted that the general follow-up numbers were significantly different according to the smoking status, as non-smoking groups had a higher number of follow-ups (p=0.01).

This study also highlighted that general and psychosocial follow-up differed according to the presence of other risk factors, and the follow-up numbers of pamong pregnant women with a risk of two and above were higher in both follow-ups (respectively, p= 0.04, and p= 0.03).

When the status of taking vitamin and iron supplementation was evaluated, it showed that both general and psychosocial follow-up among pregnant women who did not receive support were lower (p=0.01). Similarily follow-up among unvaccinated pregnant women were lower than the vaccinated groups (p=0.03). The study also noted that the number of psychosocial follow-ups with a sign of danger during pregnancy was higher than in the other groups (p=0.01). Similarily follow up among those having complications during pregnancy were lower (p=0.01).

We also observed that follow-up among those who had miscarriage-abortion and who were still pregnant differed according to the type of delivery, and were lower than the other groups who gave birth (p=0.01). Similarly, the number of psychosocial follow-ups among those with miscarriage-abortion was lower than the other groups (p=0.01), (Table-IV).

**Table-IV:** Determination of factors affecting pregnancy follow-up levels

		Number of general follow-ups during pregnancy		Number of psychosocial follow-ups during pregnancy	
		X±s.s.	p	X±s.s.	p
Occupation	Unemployed	3.34±1.05	0.03*	1.08±0.5	0.19
	Working	3.70±1.02		1.13±0.48	
Education	High school and below	3.65±0.88	0.16	0.91±0.38	0.13
	University	3.55±1.07		1.14±0.49	
Smoking	Never smoked	3.80±0.98	0.01*	1.1±0.49	0.22
	Quit during pregnancy	3.19±1.57		1.19±0.54	
	Smoking	3.23±1.15	1±0
Rh incompatibility	No	3.56±1.05	0.55	1.1±0.5	0.37
	Yes	3.61±0.99		1.18±0.39	
Disease	No	3.49±1.05	0.15	1.02±0.36	0.02*
	1	3.76±1.1		1.29±0.52	
	2 and more	3.69±0.92	1.36±0.76
Medication	No	3.48±1.04	0.03*	1.00±0.36	0.04*
	Yes	3.77±1.02		1.35±0.69	
Other risk factors	No	3.43±1.04	0.04*	1.04±0.4	0.03*
	1	3.46±1.14		1.12±0.42	
	2 and more	3.97±0.87	1.53±0.9
Vitamin and/or iron supplementation	No	2.75±1.60	0.01*	0.83±0.39	0.01*
	Yes	3.61±0.99		1.12±0.49	
Vaccination during pregnancy	No	3.37±1.22	0.03*	1.10±0.42	0.77
	Tetanus only	3.64±0.96		1.11±0.51	
Sign of danger during pregnancy	No	3.55±0.98	0.18	1.05±0.39	0.01*
	Yes	3.67±1.42		1.53±0.82	
Complication in pregnancy	No	3.63±0.97	0.01*	1.11±0.49	0.88
	Yes	1.89±1.54		1.11±0.33	
Mode of delivery and outcome	Normal-live birth	3.76±0.78	0.01*	1.19±0.52	0.01*
	Live birth by cesarean	3.97±0.55		1.21±0.44	
	Miscarriage and abortion	1.09±0.3		0.62±0.4	
	Pregnancy continued	1.47±0.64		1.32±0.55	

* Significant correlation at the 0.05 Level.

** Kruskall Wallis and Mann Whitney U test was conducted.

There was a very weak and positive relationship between the weight measurements of the participants and the number of general follow-up and psychosocial follow-ups (r_1_=17, r_2_= 0.19, p=0.01). It can be stated that higher number of general and psychosocial follow-ups were observed in patients who were overweight. The study also noted highly strong and positive relationship between the weeks of gestation of the participants and the number of general follow-up and psychosocial follow-ups (r_1_=0.73, r_2_= 0.74, p=0.01). A higher number of general and psychosocial follow-ups were observed in patients with higher gestational weeks, (Table-V).

**Table-V:** Number of follow-ups according to the measurements of pregnant women.

		Number of general follow-ups during pregnancy	Number of psychosocial follow-ups during pregnancy
Age (Years)	r	0.10	0.03
	p	0.12	0.59
Height (Centimeter-cm)	r	-0.01	-0.03
	p	0.89	0.61
Weight (Kilogram-kg)	r	0.17*	0.19*
	p	0.01	0.01
Body mass index (kg/m²)	r	0.06	-0.02
	p	0.36	0.80
Total week of pregnancy	r	0.73*	0.74*
	p	0.01	0.01

* Significant difference at 0.05 level.

**Pearson correlation test was performed.

## DISCUSSION

Our study showed that miscarriage, abortion, and other complications were less with an increase in the number of general and especially psychosocial follow-ups before and during pregnancy. In terms of healthy women’s life and reproductive health, prenatal care and follow-up during pregnancy are important. For this reason, the health of every woman who plans to give birth and enters the pregnancy process should be encouraged, and all kinds of biopsychosocial and cultural support and counseling should be provided. A quality pregnancy follow-up requires holistic, comprehensive, and continuous health care. For such care, a follow-up that takes into account the pregnant woman and her environment is required. In this follow-up, the family physician, who knows the pregnant woman individually in terms of health conditions, plays an important role. In this follow-up, modern and advanced physical conditions in the care and follow-up process as well as the individual experience of the family physician increase the quality of care during follow-ups.

It should be taken into account that quality pregnancy follow-up may differ in countries, and this may be due to economic reasons. Therefore, in low and middle-income countries, the quality of the service provided to pregnant women can be increased with the combination of standard and flexible components of the procedures in pregnancy follow-up.^5^ Comprehensive, holistic, and continuous care is important for the health care needed by elderly people, especially in rural areas.^6^ In an evidence-based randomized controlled study of young pregnant women, the rate of having a small-for-age baby was found to be significantly lower in the intervention groups. In addition, the outcomes of pregnancies that received with more detailed care were more positive.^7^ The study also noted that the mother tongue of the pregnant woman is also effective in the quality of life in terms of communication in physiological pregnancy.^8^

In a systematic study that stated that prenatal care models are gaining increasing importance, the results of improved care applied to opioid-dependent, adolescent, and low-income pregnant women are significant.^9^,^10^ The role of family physicians, who are ready for development and different approaches, is important for health follow-ups that require special approaches and care such as pregnancy.^11^ In contrast to traditional care methods, the positive contribution of pregnancy-focused care to maternal health and breastfeeding was emphasized.^12^

In our study, we investigated the results of biopsychosocial care contents as well as the general care follow-ups we provide to pregnant women in our modern family medicine practice, which requires pregnancy-specific approaches. For this purpose, we evaluated the data on the risks that will affect the baby’s brain development along with the care provided to the mother during general pregnancy follow-ups. In the interviews with the parents, we analyzed the results of the training on the psychological development of the baby. In our study, it was found that pregnant women were followed up at least 3.56 times in general and at least once in psychosocial follow-up. It was noted that the average of gestational weeks was 36.59% and pregnancy-related complications occurred at a rate of only 3.6%.

This study emphasized that the undesirable effects of possible risk factors decrease with appropriate follow-ups in adolescent pregnancies.^13^ In a review, it was emphasized that there are insufficient studies reporting that diseases such as obesity, diabetes, hypertension, and hyperlipidemia are associated with cardiovascular risk factors during pregnancy, and requires long-term care, and optimal treatment strategies.^14^ In another cohort study investigating the relationship between gestational age and the risk of endometrial cancer, it was shown that pregnancy reduces cancer regardless of gestational age. Therefore, encouraging pregnancy by family physicians is as important as a follow-up.^15^ In our study, we noticed that 74.2% of the pregnant women had no risk factors, 16.5% had one, and 9.3% had two or more risk factors. The fact that the amount of risk seen in pregnancy and the complications that may be related to it are low in the follow-ups suggests that their follow-up by the family physician is appropriate.

In a randomized controlled study, a questionnaire was used to ask fathers during pregnancy follow-up, and their knowledge at the end of pregnancy and delivery was measured.^16^ A similar study, aimed to educate pregnant women in the control group who received standard perinatal care with a web-based approach and in groups with added psychosocial support.^17^ Another study recommends mental health assessment for prenatal depression in parents as a risk factor.^18^

In yet another study, it was shown that pregnant women with gestational diabetes were not adversely affected by pregnancy outcomes. In such situations, the effect of psychosocial follow-up is as important as the personal situation of the pregnant woman.^19^ Similarly, preventive medicine and a holistic psychosocial approach are as important for the quality of pregnancy as for treatment in patients with urinary incontinence.^20^ Although gynecological methods and the individual characteristics of the pregnant women are important in cases such as infertility, psychosocial follow-ups are complementary before and during pregnancy.^21^

These applications are very appropriate and should be used in modern pregnancy follow-ups. In our family medicine, face-to-face meetings with the father standard information processes are included in the psychosocial monitoring of pregnancy. In all of our pregnant follow-ups, the father is interviewed at least once, and after that, we stay in touch and provide support to the father and the pregnant woman regarding pregnancy complications and mental health.

In a study conducted in China, it was shown that health services increase with economic growth, but mental health services and quality care during pregnancy are lacking. It was pointed out that due to the risk of social stigma, help from family and friends is easier to reach instead of mental health professionals.^22^ However, family physicians, who are very close to the family, are ideal for this job and these problems can be solved with standardized pregnancy monitoring and psychosocial care can be added. Psychosocial support designed during pregnancy and postpartum is recommended as an effective intervention for pregnant women and mothers.^23^

In our study, results such as the number of pregnant follow-ups, gestational week duration, and live birth rates were at the desired level. In addition, miscarriage, abortion, and other complications were found to be statistically significantly less in all pregnancies followed up. In addition to holistic, comprehensive, and continuous care, an improved psychosocial approach is important in the positive results of pregnancy follow-ups in our family medicine practice.

Researchers in one of the studies has advocated that all of the additional examinations, tests, vaccinations, treatments, and interventions should be taken into account in addition to prenatal care four times during pregnancy.^24^ This is a very appropriate and important suggestion. We implemented this in our study as family medicine is the appropriate environment for this recommendation. In addition, we suggest that the quality of health in pregnancy follow-ups will increase with the psychosocial care we have developed in addition to standard care with a holistic, comprehensive, and continuous care approach in our family medicine practice.

### Limitations of the study:

The data of the pregnant women, who had a lack of information during the study period and who went out of the region for any reason could not be used.

## CONCLUSION

Our study showed that miscarriage, abortion, and other complications are very rare with the increase in the number of general and especially psychosocial follow-ups before and during pregnancy. Pregnancy follow-ups carried out by family physicians with developed guidelines are important for the health of the mother and baby.
